# Fear of childbirth, anxiety and depression in three groups of primiparous pregnant women not attending, irregularly attending and regularly attending childbirth preparation classes

**DOI:** 10.1186/s12905-020-01048-9

**Published:** 2020-08-14

**Authors:** Robab Hassanzadeh, Fateme Abbas-Alizadeh, Shahla Meedya, Sakineh Mohammad-Alizadeh-Charandabi, Mojgan Mirghafourvand

**Affiliations:** 1 Midwifery Department, Tabriz University of Medical sciences, Tabriz, Iran; 2 Reproductive Health Research Center, Tabriz University of Medical Sciences, Tabriz, Iran; 3 South Asia Infant Feeding Research Network (SAIFRN), School of Nursing, Faculty of Science, Medicine and Health, University of Wollongong, Wollongong, Australia; 4 Department of Midwifery, Faculty of Nursing and Midwifery, Tabriz University of Medical Sciences, Tabriz, Iran; 5 Social Determinants of Health Research Center, Tabriz University of Medical Sciences, Tabriz, Iran

**Keywords:** Childbirth fear, Anxiety, Depression, Childbirth preparation classes

## Abstract

**Background:**

Lack of knowledge and fear of the unknown during pregnancy and childbirth make mothers fearful, worried, and anxious. Maternal fear and anxiety can lead to problems such as preterm childbirth and low birth weight. Increasing women’s knowledge through prenatal education can prepare them for childbirth and improve their health. The present study was conducted to compare fear of childbirth, anxiety and depression during pregnancy in three groups of primiparous pregnant women who were either not attending, irregularly attending, or regularly attending childbirth preparation classes.

**Methods:**

A total of 204 primiparous pregnant women attending health centers in Tabriz, Iran, were selected by cluster sampling and assigned to the following three groups: Not attending, irregularly attending (attending one to three sessions of classes) and regularly attending (attending four to eight sessions of classes). Childbirth fear, pregnancy anxiety and depression questionnaires were completed for them through interviews. The general linear model was used to compare their fear of childbirth and prenatal anxiety and depression.

**Results:**

According to the general linear model, the scores of fear of childbirth (*p* <  0.001), anxiety (*p* <  0.001) and depression (*p* = 0.006) were significantly lower in the group of pregnant women regularly attending the classes compared to the non-attending group of women. No significant differences were observed between the regularly-attending and irregularly-attending groups in terms of fear of childbirth (*p* = 0.066), anxiety (*p* = 0.078), and depression (*p* = 0.128).

**Conclusion:**

Prenatal training can reduce fear, anxiety and depression in primiparous women. Incorporating such training into prenatal care helps improve maternal health.

## Background

Pregnancy is one of the most important events and unforgettable moments in women’s life that, despite being associated with many positive feelings [1], can also be one of the most stressful events for them. Pregnancy has been referred to as an emotional crisis for some women; and if not properly managed, it can lead to maternal and neonatal complications [2]. There is a lot of evidence suggesting that many psychological problems, including experiencing fear, anxiety and depression are associated with pregnancy [1].

Fear during pregnancy is manifested as fear of miscarriage, fear of fetal abnormalities, and fear of not being a good mother. Pregnant women’s fear heightens toward the end of pregnancy, mostly due to fear of childbirth and delivery pain [1]. Lack of knowledge and fear of the unknown during pregnancy and childbirth make mothers worried and anxious. Maternal fear, anxiety [3], and depression are connected to problems such as preterm childbirth and low birth weight. Furthermore, neonates born of highly fearful and anxious mothers are also likely to have weak immune systems [1].

Due to the increasing rates of cesarean delivery across the world, international policies tend to encourage vaginal delivery [4–6], and various philosophies and approaches are employed to encourage vaginal delivery [7]. The Lamaze philosophy of birth became popular in the United States in the 1960s as an approach that supports educating mothers to have active participation in childbirth and also childbirth without unnecessary interventions [8]. In some studies, women attending childbirth preparation classes were better adapted to labor pain, had fewer medications administered to them in labor, and needed less operative delivery [9–11].

In a study conducted by Stamler, pregnant women attending prenatal educational classes stated that increased knowledge due to participation in these classes had reduced their anxiety and fear. The women in study reported the relaxation techniques very helpful. Moreover, they described the atmosphere of these classes such that they could comfortably ask their questions and receive answers [4]. The results obtained by Lee & Holroyd showed that pregnant women attending childbirth preparation classes were better able to manage their pregnancy, childbirth and postpartum period compared to women who merely received routine care [5].

In the past, prenatal care in Iran was confined to regular examinations, routine tests, and ultrasounds. This program, however, was not adequate for mothers, and their lack of knowledge and preparation led to anxiety and complications followed by daily increase in medical interventions [6]. Accordingly, in 2008, the Ministry of Health and Medical Education started holding classes known as ‘physiological childbirth preparation classes’. These classes are held for all pregnant woman in eight sessions during weeks 20 to 37 of pregnancy. The content of classes includes the anatomy of the female reproductive system, body adaptations during pregnancy, fetal growth and development, prenatal care, nutrition, personal hygiene and mental health, risk factors during pregnancy, benefits of vaginal delivery, pain relief methods, postpartum examinations, abnormal symptoms and neonatal nursing [7]. From the time these classes were first held to the present day, only a few studies have been conducted in Iran on the relationship between attending different childbirth preparation classes and fear of childbirth, maternal depression and anxiety [8–10]. The present study is the first study that assesses the effect of national childbirth classes held by the Ministry of Health on prenatal fear, anxiety and depression in three groups of women: a) regular attenders, b) irregularly attenders, and c) not attenders.

## Methods

### Study design and participants

The present study is part of a mixed method research whose protocol has been previously published [11]. This part of the study has been cross-sectional and examines a population consisting of 204 primiparous pregnant women with a gestational age of 35–37 weeks that their attendance was used to create three categories, defined as regular attendance (attending four to eight sessions of educational classes), irregular attendance (attending one to three sessions) and non-attendance groups. According to the training guide of preparation classes for midwives in Iran, mothers who attend classes for 1–3 sessions are not considered trained, so the division was based on this [7].

The study inclusion criteria consisted of living in Tabriz city, being primiparous, and having a gestational age of 35–37 weeks. The study exclusion criteria consisted of having a multiple pregnancy, being multiparous, having preterm or post-term birth, non-cephalic presentation, history of physical problems, history of depression, use of medications inducing depression symptoms, stressful event in the family and obstetrics problems.

The sample size was determined according to the score of fear of childbirth using G-power. Based on the results of a study conducted by Najafi et al. [10] on the relationship between attending childbirth preparation classes and fear of childbirth, and considering M_1_ = 37.29 (mean score of fear in the routine care group), SD_1_ = 9.55, M_2_ = 32.30 (mean score of fear in the group attending classes), SD_2_ = 9.31, one-sided α = 0.05, and power = 90%, the sample size per group was found as 62, which was increased to 68 to take account of a potential attrition of 10%, making the total sample size 204.

### Sampling

**Fig. 1:**
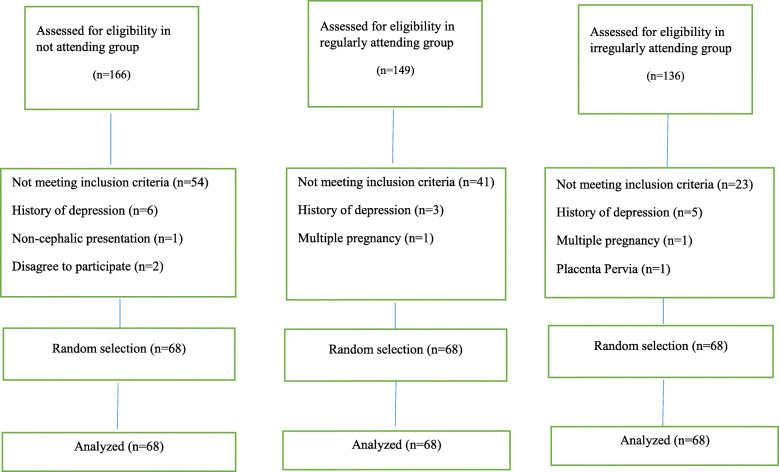
Flowchart of the study

Cluster sampling was performed, and out of the 20 healthcare complexes in Tabriz, seven were selected through simple random method, and sampling was performed in all the health centers covered by each of the selected complexes (each complex covered four to five health centers, and sampling was conducted in a total of 29 centers). Eligible pregnant women were selected from each center from the primiparous pregnant women who had a gestational age of 35–37 weeks. Based on the published study protocol [11] and for more generalizability of research findings, random sampling was used to select of 68 participants from the eligible pregnant women in all three groups (Fig. 1). After the inclusion of these eligible women, the study objectives and methods were fully explained to them and written informed consent was then obtained from those willing to take part, and the questionnaires were completed for them through interviews.

### Data collection tools

In the present study, data were collected using demographic and obstetric questionnaires, the Wijma Delivery Expectancy/Experience Questionnaire (W-DEQ) – Version A, the Pregnancy-Related Anxiety Questionnaire (PRAQ) by Van den Bergh, and the Edinburgh Perinatal Depression Scale (EPDS). The Persian version of all instruments used in this study have been validated in Iran after obtaining license from the copyright holders of the English version of the tools [12–14].

The demographic questionnaire included items on age, age at onset of sexual life, education, occupation, BMI, spouse’s age, education, and occupation, spouse’s support and marital satisfaction. Spouse’s support and marital satisfaction were assessed with a subjective item and responses were based on 5 point Likert scale including very high, high, moderate, low and very low and participants could choose one of the options.

The obstetric questionnaire inquired about history of miscarriage, infertility, unwanted pregnancy, and fetal gender preference and its match with the actual fetal gender. The validity of this questionnaire was confirmed in the present study through content and face validity methods.

The W-DEQ was developed by Wijma et al. in 1998 and measures prenatal fears and expectations with 33 items. Mothers specify their personal feelings and cognition based on a six-point Likert scale (from ‘never’ =0 to ‘very often’ =5). The total score of this questionnaire is the sum of the scores of all the items, and ranges from 0 to 165, and higher scores suggest greater fear of childbirth. Items 2, 3, 6, 7, 8, 11, 12, 15, 19, 20, 24, 25, 27, and 31 are scored in reverse. Wijma et al. determined the reliability of the questionnaire by the split-half method (0.89) and Cronbach’s alpha coefficient (0.92) [15]. Mortazavi reported the reliability of this questionnaire in Iran with Cronbach’s alpha of 0.91 [12]. In the present study, the reliability of this questionnaire was confirmed with a Cronbach’s alpha of 0.86.

The PRAQ was introduced by Van den Bergh in 1999 and contains 34 items in five domains [16]. Huizink et al. conducted a factor analysis and identified the domains with high factor loading and categorized the five domains into three, as follows: 1- Fear of giving birth; 2- Worries about bearing a physically or mentally handicapped child; and 3- Concern about own appearance. They reduced the items from 34 to ten. The scoring of this questionnaire is based on the Likert scale, with “Definitely True” scoring five points and “Definitely Not True” scoring one point. Higher scores indicate higher anxiety in the respondent. Huizink et al. argued that this 10-item PRAQ has acceptable face and content validities. They reported the reliability of the ‘fear of giving birth’, ‘worries about bearing a physically or mentally handicapped child’, and ‘concern about own appearance’ subscales with scores of 0.83, 0.87, and 0.83, respectively [17]. In Iran, the psychometric assessment of this questionnaire was carried out by Karamouzian et al., and its reliability was reported as 0.78 [13]. In the present study, the reliability of this questionnaire was confirmed with a Cronbach’s alpha of 0.82.

The EPDS measures prenatal and postnatal depression and was developed by Cox et al. in 1987. This scale consists of ten four-option items, some of which are organized from low to high severity (items 1, 2, and 4), and some from high to low (items 3, 5, 6, 7, 8, 9, and 10). Each item scores from zero to three points based on the severity of the symptom, and the respondent’s score is the sum of the scores of all ten items and can vary from zero to 30. Women scoring higher than the threshold of 12 have depression with varying severities [18]. In Iran, the psychometric assessment of this questionnaire was performed by Montazeri et al. [14], and in the present study, the reliability of the questionnaire was confirmed with a Cronbach’s alpha of 0.81.

### Data analysis

After completing the questionnaires, data were analyzed in SPSS-21. The normality of the quantitative data was assessed using the Kolmogorov-Smirnov test. The homogeneity of the study groups in terms of demographic details was assessed using the one-way ANOVA, the Chi-squared test, Chi-squared for trend test, and Fisher’s exact test. The study groups were compared in terms of prenatal fear, anxiety and depression using the one-way ANOVA in the bivariate analysis and the general linear model with the adjustment of certain demographic variables in the multivariate analysis. LSD Post-hoc test was used for multiple comparison after it. In general linear model, fear of childbirth, anxiety and depression were considered as dependent variables and study groups were considered as independent variables and spouse’s education, income, occupation, spouse’s occupation, spouse’s support, and marital satisfaction were considered as confounding variables. *p* <  0.05 was taken as the significance level.

## Results

No significant difference was found between the study groups in terms of demographic details including woman’s age and education, spouse’s age, age at the onset of sexual life, BMI, wanted pregnancy and gestation period (*p* > 0.05), but there were significant differences between the three groups in terms of woman’s occupation, spouse’s education and occupation, income, spouse’s support, and marital satisfaction (*p* <  0.05). The mean (standard deviation) age was 25.8 (5.7) years in the non-attending group, 27.0 (5.6) years in the irregularly-attending group, and 25.7 (4.7) years in the regularly-attending group (*p* = 0.297). The majority of the women in the non-attending, irregularly-attending, and regularly-attending groups (76.5, 66.5, and 77.9%, respectively) had adequate incomes. Less than half of the women in the non-attending group (41.9%) had high school diploma, while 52.9% of the women in the irregularly-attending group and 41.9% of those in the regularly-attending group had university education. The majority of the women in the non-attending, irregularly-attending, and regularly-attending groups (97.1, 76.5, and 82.3%, respectively) were housewife. About one-third of the spouses in the non-attending group (36.8%) had high school diploma, while half of the spouses in the irregularly-attending (50%) and regularly-attending (55.8%) groups had university education. The majority of the women in the non-attending, irregularly-attending, and regularly-attending groups (88.2, 89.7, and 88.2%, respectively) had wanted pregnancies (Table 1).

**Table 1:** Characteristics of the study participants (*n* = 204)

Variable	Not attending Mean (SD)	Regularly-attending Mean (SD)	Irregularly-attending Mean (SD)	*p*-Value
Age (years)	25.8 (5.7)	25.7 (4.7)	27.0 (5.6)	0.297^*^
Spouse’s Age	30.9 (4.2)	30.8 (4.2)	32.0 (5.0)	0.212^*^
Age at the onset of sexual life	23.1 (5.4)	22.8 (4.6)	23.9 (5.2)	0.448^*^
Education				
Illiterate	2 (2.9)	1 (1.5)	1 (1.5)	0.083^†^
Elementary	2 (2.9)	2 (2.9)	1 (1.5)	
Intermediate	9 (13.3)	8 (11.8)	10 (14.7)	
High school	9 (13.3)	11 (16.2)	9 (13.3)	
Diploma	28 (41.2)	18 (26.5)	11 (16.2)	
College	18 (26.5)	28 (41.2)	36 (52.9)	
Occupation				
Housewife	66 (9.7)	56 (82.3)	52 (76.5)	0.001^†^
Employee	2 (2.9)	8 (11.8)	15 (23.1)	
University student	0	4 (5.9)	1 (1.5)	
Income				
Not at all sufficient	10 (14.7)	2 (2.9)	6 (8.8)	0.014^†^
Relatively sufficient	52 (76.5)	53 (77.9)	45 (66.2)	
Completely sufficient	6 (8.8)	13 (19.1)	17 (25.0)	
Spouse’s Education				
Illiterate	3 (4.4)	0	1 (1.5)	0.001^‡‡^
Elementary	7 (10.3)	2 (2.9)	5 (7.4)	
Intermediate	11 (16.2)	5 (7.4)	3 (4.4)	
High school	9 (13.3)	4 (5.9)	4 (5.9)	
Diploma	25 (36.8)	19 (27.9)	21 (30.9)	
College	13 (19.1)	38 (55.8)	34 (50.0)	
Spouse’s occupation				
Unemployed	2 (2.9)	2 (2.9)	1 (1.5)	0.045^†^
Employed	8 (11.8)	19 (27.9)	13 (19.1)	
Worker	25 (36.8)	10 (14.7)	16 (23.5)	
Free Job	33 (48.5)	37 (54.4)	38 (55.8)	
Spouse’s support				
Very high	20 (29.4)	41 (72.1)	34 (50.0)	> 0.001^†^
High	22 (32.4)	16 (23.5)	20 (29.4)	
Moderate	24 (35.3)	10 (14.7)	14 (20.6)	
Low	1 (1.5)	0	0	
Very Low	1 (1.5)	1 (1.5)	0	
Marital Satisfaction				
Very high	20 (29.4)	49 (60.3)	37 (54.4)	> 0.001^§^
High	27 (39.7)	12 (17.6)	19 (27.9)	
Moderate	20 (29.4)	6 (8.8)	12 (17.6)	
Low	1 (1.5)	1 (1.5)	0	
Unwanted sex of baby	14 (20.6)	17 (25.0)	20 (29.4)	0.622 ^‡^
Unwanted pregnancy	8 (11.8)	9 (13.2)	7 (10.3)	0.895^‡^

^*^One- Way ANOVA, ^†^Fisher’s Exact Test, ^‡^ Chi-square test, ^§^Linear-by-Linear Association

According to the one-way ANOVA, the mean (standard deviation) fear score was 40.4 (19.2) in the regularly-attending, 48.7 (20.5) in the irregularly-attending, and 62.6 (18.8) in the non-attending group, and their mean (standard deviation) anxiety score was 22.8 (8.0), 25.9 (7.9), and 29.4 (6.9) respectively, and their mean (standard deviation) depression score was 6.1 (4.1), 7.4 (4.3), and 9.3 (5.0), respectively. The mean score of fear of childbirth in the regularly-attending group (*p* < 0.001) and the irregularly-attending group (*p* < 0.001) was significantly lower compared to the non-attending group. Also, the mean score of anxiety in the regularly-attending (*p* < 0.001) and irregularly-attending (*p* = 0.007) groups was significantly lower compared to the non-attending group. Similarly, the mean score of depression in the regularly-attending (*p* < 0.001) and irregularly-attending (*p* = 0.016) groups was significantly lower compared to the non-attending group (Table 2).

**Table 2:** A comparison of the scores of fear of childbirth and prenatal anxiety and depression in groups based on the ANOVA

Variable	N-A* n = 68	R-A^†^ *n* = 68	I-A^‡^ *n* = 68	Comparison between groups (*p*-Value) ^‡‡^ N-A with R-A N-A with I-A I-A with R-A		
	Mean (SD)	Mean (SD)	Mean (SD)			
Fear of childbirth	62.8 (11.8)	40.4 (19.2)	48.7 (20.5)	< 0.001	< 0.001	0.014
Anxiety	29.4 (6.9)	22.8 (8.1)	25.9 (7.9)	< 0.001	0.007	0.022
Depression	9.3 (5.0)	6.1 (4.1)	4.7 (4.3)	< 0.001	0.016	0.097

*Not-attending group, ^†^Regularly-attending group, ^‡^Irregularly-attending group

^‡‡^LSD post-hoc test was used

After the adjustment of spouse’s education, income, occupation, spouse’s occupation, spouse’s support and marital satisfaction, the general linear model results showed that the mean scores of fear of childbirth (*p* < 0.001), anxiety (*p* < 0.001), and depression (*p* = 0.006) in the regularly-attending group were significantly lower than those in the non-attending group, but no significant difference was found between the regularly-attending and irregularly-attending groups in terms of fear of childbirth (*p* = 0.066), anxiety (*p* = 0.078), and depression (*p* = 0.128) (Table 3).

**Table 3:** Comparison of the scores of fear of childbirth, anxiety and depression in the non- attending, irregularly- attending and regularly- attending groups based on the general linear model

Variable	B*	95%Confidence Interval	***p***-Value
**Fear**			
Regularly attending (Reference)	0		
Irregularly attending	6.4	−0.4-13.2	0.066
Not attending	19.2	11.8–26.6	< 0.001
**Anxiety**			
Regularly attending (Reference)	0		
Irregularly attending	2.5	−0.2- 5.3	0.078
Not attending	6.4	3.4–9.4	< 0.001
**Pregnancy depression**			
Regularly attending (Reference)	0		
Irregularly attending	1.2	0.3–2.7	0.128
Not attending	2.3	0.6–4.0	0.006

*Values have been adjusted for spouse’s education, income, occupation, spouse’s occupation, spouse’s support, and marital satisfaction

## Discussion

After the adjustment of spouse’s education, income, occupation, spouse’s occupation, spouse’s support and marital satisfaction, the results showed that the mean scores of fear of childbirth, anxiety, and depression were significantly lower in the group regularly attending childbirth preparation classes than those in the non-attending group, but no significant difference was found between the regularly-attending and the irregularly-attending groups.

In the present study, the mean scores of fear of childbirth were significantly lower in the group regularly attending childbirth preparation classes than those in the non-attending group. In a quasi-experimental study conducted in Turkey by Gokce Isbir et al. the mean score of fear of childbirth was lower in the intervention group than in the control group [19]. In a prospective cohort study conducted by Najafi et al. on 202 primiparous pregnant women divided into two groups of willing and unwilling to attend childbirth preparation classes, a significant difference was observed in fear scores between the two groups in the third trimester and after attending classes, as the mean score of fear of childbirth was lower in the attending group than that in the routine care group after the end of the classes [10]. These results agree with the present findings. Fear of childbirth is a serious issue for women that can make mothers avoid pregnancy, cause maternal and fetal stress, and increase mothers’ requests for cesarean section [20]. Childbirth preparation classes reduce mothers’ fears by increasing their knowledge of pregnancy, childbirth and the postpartum period and instilling confidence in them about their ability to tolerate the pain of labor [3, 21]. In the present study, decrease of anxiety and depression by attending in childbirth preparation classes could be one of the reasons for the decrease in the fear of childbirth because according to the results of studies, both depression and anxiety have been increased the prevalence of fear of childbirth [22, 23] and other reason could be increasing pregnant women knowledge about pregnancy and childbirth by attending in Childbirth preparation classes.

In the present study, the mean score of anxiety was significantly lower in the group regularly attending childbirth preparation classes than in the non-attending group, but no significant difference was observed between the regularly-attending and irregularly-attending groups. In an interventional study conducted by Firouzbakht et al. holding prenatal training classes by the researcher helped reduce hospital anxiety, and the mean score of anxiety was lower in the trained group than the non-trained group [8]. In a study by Stamler (1998), the participants in the prenatal training classes stated that the increased knowledge resulting from participation in these classes had reduced their anxiety and fear. Furthermore, they considered relaxation techniques taught in these classes very helpful and described the atmosphere of these classes such that they could comfortably ask questions and receive answers [4]. In a study conducted by Lee et al. (2009) in China, childbirth preparation classes reduced mothers’ anxiety and increased their tolerance of labor pain [5]. In a study by Koehn (2002), prenatal training reduced anxiety [24]. The results of all these studies concur with the present findings. Teaching topics such as changes during pregnancy, signs of onset of labor and non-medicinal pain relief techniques can help reduce women’s prenatal anxiety [3, 5, 21].

In the present study, the mean score of depression was significantly lower in the group regularly attending childbirth preparation classes compared to the non-attending group, but no significant difference was found between the regularly-attending and irregularly-attending groups. In a study conducted by Toohill et al. on 339 pregnant women with severe fear divided to a telephone counseling training group and a control group, training reduced their fear of childbirth and prenatal depression [25]. In the study by Mousavinejad et al. (2015), teaching pregnant women cognitive skills was an effective method for reducing prenatal anxiety and depression [26]. Prenatal training mentally prepares women for childbirth [21, 24].

In the present study, no significant difference was found between the regularly-attending and irregularly-attending groups in terms of the mean scores of fear, anxiety and depression. The results of studies have shown that shorter training sessions were also effective in reducing fear of childbirth and anxiety [5, 27, 28]. Therefore, pregnant women should be encouraged to attend as many sessions as they can if they are not able to not attend all the sessions.

Evidence demonstrated that training women can increase their confidence about their ability to deal with childbirth and the pain of labor, reduce medical interventions during childbirth, reduce medical costs, and improve maternal health [25]. The Progetto Obiettivo Materno Infantile (POMI: Program for Mother and Child Health) specifies prenatal training as a primary prevention and health measure, and given the results of previous studies, incorporating prenatal training into prenatal care can be effective in improving maternal health [29].

### Limitations and strengths

The main limitation of this study is that the participants in this study consisted of primiparous pregnant women from a healthcare complexes from Tabriz city, Therefore, the results couldn’t be generalized to multiparous women or primiparous pregnant women from other cities. Also, the questionnaires were completed only by 68 participants who selected through random sampling. Thus, statistical analysis wasn’t performed for all eligible pregnant women in all three groups. Another limitation is that this study was not a randomized trial and childbirth educational classes were not held by the researcher team. Also, the women with history of depression and stressful events in this study were excluded that can decrease the generalizability of the results to these women. Despite these limitations, this study has several strengths such as random sampling and this is the first study to our knowledge to investigate the effects of childbirth preparation classes in Tabriz city-Iran.

## Conclusion

The results of this study revealed benefits of participation in the childbirth preparation classes, including a reduction in fear of childbirth, anxiety and depression during pregnancy. Therefore, participation in this classes should be seriously considered as a component of standard prenatal care.

## Data Availability

The datasets used and analysed during the current study are available from the corresponding author on reasonable request.

## Abbreviations

W-DEQ: Wijma delivery expectancy/experience questionnaire
PRAQ: Pregnancy-related anxiety questionnaire
EPDS: Edinburgh perinatal depression scale
ANOVA: Analysis of variance
LSD: Least significant difference
